# Disability and Its Influencing Factors among the Elderly in a County, Guangxi Province, China

**DOI:** 10.3390/ijerph15091967

**Published:** 2018-09-09

**Authors:** Shiyi Chen, Jian Qin, You Li, Yi Wei, Bingshuang Long, Jiansheng Cai, Jiexia Tang, Xia Xu, Guoqi Yu, Zhiyong Zhang

**Affiliations:** 1 Department of Occupational and Environmental Health, School of Public Health, Guangxi Medical University, Shuangyong Road No.22, Nanning 530021, China; chenshiyi@stu.gxmu.edu.cn (S.C.); gxmucommoner@163.com (J.Q.); weiyi@stu.gxmu.edu.cn (Y.W.); longbingshuang@stu.gxmu.edu.cn (B.L.); caijiansheng@stu.gxmu.edu.cn (J.C.); tangjiexia@stu.gxmu.edu.cn (J.T.); hsuxia0514@163.com (X.X.); gqyu@stu.gxmu.edu.cn (G.Y.); 2 Department of Occupational and Environmental Health, Faculty of Public Health, Dali University, Dali 671000, China; liyou121300@163.com

**Keywords:** elderly, disability, influencing factors

## Abstract

*Objectives*: This study aims to understand the disability status of the elderly residents of a County (Guangxi Province, China) and explore its influencing factors. *Methods*: Respondents consisted of 2300 elderly people aged 60 and above from three townships in the county we studied. The Activities of Daily Living (ADL) Scale was used to assess the disability of the elderly sample. Chi-square test was applied to compare the disability rate among the elderly with different demographic characteristics. The graph showed the disability rates of ADL, six items of Physical Activities of Daily Living (PADL) and eight items of Instrumental Activities of Daily Living (IADL) at different ages. Binary logistic regression was used to analyze the influencing factors of disability rate among the elderly. *Results*: The disability rates of ADL, PADL, and IADL in the elderly were 43.4%, 11.6%, and 42.4%, respectively. As with the increase in age, the disability rates of ADL, IADL, PADL, and their 14 items gradually increased (*p* < 0.05), with walking, using the telephone, and using public vehicles having higher disability rates than other items. The influencing factors of ADL disability were gender (OR = 0.579, 95%CI = 0.441–0.759), age (OR = 2.270, 95%CI = 1.867–2.759; OR = 4.719, 95%CI = 2.998–7.429; OR = 6.249, 95%CI = 3.667–10.648), educational level (OR = 2.844, 95%CI = 2.076–3.897; OR = 1.677, 95%CI = 1.246–2.230), and having metabolic syndrome (MetS) (OR = 1.298, 95%CI = 1.044–1.613). Compared with ADL, the influencing factor of PADL disability was gender, whereas that of IADL disability was whether someone had MetS. *Conclusions*: With age, the possibility of ADL, PADL, and IADL damage in the elderly is higher. Gender, age, educational level, the number of chronic diseases, and whether someone has MetS might be the influencing factors of disability. Interventions should be taken from a variety of sources specific to the content of each entry.

## 1. Introduction

By the end of August 2015, the Geriatric Society of China announced that Guangxi was the largest province in China with a population exhibiting good longevity rates [1]. Wang et al. [2] also studied regional longevity in China and reported Hechi City as one of the areas with high longevity levels in China. Hechi is a relatively underdeveloped area in Guangxi and the Zhuang people comprise its main ethnicity. The Bama Yao Autonomous County of Hechi is recognized as the “hometown of longevity in the world” and has aroused widespread global interest [3,4]. However, other counties and cities in the Hechi area, which has six “longevity towns,” do not enjoy the same level of popularity (Bama Yao Autonomous County, Donglan County, Fengshan County, Yizhou City, Dahua Yao Autonomous County and Tiane County). The county we studied belongs to Hechi and is adjacent to Bama and is one of the six “longevity towns,”. The area is located in the northwest of Guangxi, the southern margin of the Yunnan-Guizhou plateau, in the middle reaches of Hongshui River. To date, no research has been conducted on the health status of the elderly in the county.

According to estimates, by 2050, the population of people aged 80 around the world would reach 400 million, 70% of whom can be found in the underdeveloped regions [5]. Based on the data released by the National Bureau of Statistics of China, in 2016, the number of elderly population aged 60 and above has increased to nearly 231 million, accounting for 16.7% of the total population; meanwhile, the elderly population aged 65 and above has reached 150 million, accounting for the total population 10.8% [6]. Recent estimates indicate that the proportion of Chinese adults aged 80 and above will increase from 11.5% in 2015 to 23.2% in 2050 [7]. With the increasing number of elderly population in China, the proportion of those in rural areas accounts for about 70% of this population [8]. Compared with China’s cities, rural areas, which are relatively backward in culture and economy, are being threatened by environmental degradation, declining schedule activities and chronic diseases. The burden on old-age security for the elderly in rural areas will be more important than ever before [9,10]. Thus, the problem of ageing in rural areas is worthy of public attention than that in urban areas.

With the development of the ageing population, the health condition of the elderly and the major influencing factors have attracted significant scholarly attention. Dealing with the risks and burdens of disability caused by ageing is crucial. The ability of daily life (ADL) refers to the most basic and common body movements that people must perform repeatedly every day to live independently [11]. ADL consists of physical activities of daily living (PADL) and instrumental activities of daily living (IADL) [12]. Several studies [13,14,15] found significant differences in ADL disability rates among the elderly in different regions. For example, a survey conducted by the Mexican Health and Ageing Research Center (MHAS) for older Mexicans aged 80 years and older found that in the 2000–2001 and 2010–2012 cohort studies, the MHAS ADL disability rates increased from 37.5% to 44.0%, indicating that, as time goes by, the disability rate of ADL increased as well [13]. A survey of 23,815 elderly people in Brazil with an average age of 69.8 years reported an ADL disability rate of 30.1% [14]. A 2002 study on the ADL disability rates of the elderly in China revealed the disability rates of 6.9%, 23.6%, and 42.7% among the age groups of 65–79, 80–89, and 90–99 years old, respectively (total disability rate: 41.0%) [15]. At present, although many studies on ADL disability have been published, most have examined overall ADL or IADL disability. Few studies have analyzed disability from the overall effect on each item. Hence, the purpose of the current study is to investigate the possible influencing factors of disability (ADL, PADL, IADL, and 14 items) among the elderly people aged 60 and above, who are living in the county we studied (Guangxi Province, China).

## 2. Methods

### 2.1. Study Population

From June 2016 to July 2017, we recruited 2300 elderly people aged 60 and above from three townships in a County (Guangxi Province, China). Of all the towns in the County, according to the proportion of the aged over 60 in the total population of the town, we chose the top three of the above proportion as the research site. All the older persons over 60 years of age in these three townships were the subjects of this study. The inclusion criteria were as follows: permanent residents with formal household registration and those who were aged 60 years or above at the time of the survey. The exclusion criteria are as follows: people with a mental system disease, Alzheimer’s, or severe cognitive impairment; those who refused investigation and physical examination; and those who lived beyond the areas being investigated.

The project was initiated by obtaining data from the respondents aged 60 and above from the local Department of Health. The elderly were guided to the local hospital of the town under the care of village doctors and cadres. After obtaining consent, the investigators administered the questionnaire and conducted health examinations on the elderly. The investigators consisted of trained staff and physical examination doctors. If the elderly cannot complete the above tasks independently, family members assisted them in completing the relevant questionnaire survey and physical examination.

### 2.2. Activities of Daily Living (ADL) Scale

The ADL Scale was adapted from the 1969 study of American scholars Lawton and Brody [12]. The ADL Scale consists of 14 items to assess disability and is divided into two parts: Physical Activities of Daily Living (PADL) and Instrumental Activities of Daily Living (IADL). The six factors of PADL are eating, toileting, clothing, grooming, walking, and bathing and the eight factors of IADL are cooking, shopping, making phone calls, doing housework, washing clothes, using vehicles, taking medicine, and handling money and goods. Each item has four options, and the scores range from 1 to 4: (1) I can do it completely, (2) I have some difficulties, (3) I need help, and (4) I cannot do it at all. The total score is 14–56, with the lowest score of 14 indicating a normal level and a score of 15 or above indicating ADL disability. In terms of individual item score, a score of 1 indicates a normal level, scores of 2–4 indicate disability, a PADL score >6 indicates disability, and an IADL score >8 indicates disability [16]. The scale has good applicability in rural areas [17] and the Cronbach’s redundancy coefficient is 0.811.

### 2.3. Metabolic Syndrome (MetS) Criterion

Metabolic syndrome (MetS) is a group of risk factors for cardiac metabolic disorders including abdominal obesity, dyslipidemia, hypertension and hyperglycemia [18]. According to the actual situation of the Chinese population, we applied the International Diabetes Federation Criteria [19] to define MetS as follows: judging from any three or more of the following five factors of waist circumference, blood pressure, triglycerides, high-density lipoprotein cholesterol, and fasting blood glucose. The specific standards included the following: (1) waist circumference: male > 90 cm, female > 80 cm; (2) blood pressure: systolic blood pressure > 130 mmHg or diastolic blood pressure > 85 mmHg; (3) triglycerides: at least 1.7 mmol/L or under treatment; (4) HDL: male < 1.03 mmol/L, female < 1.29 mmol/L or receiving related treatment; and (5) fasting blood glucose: at or above 5.6 mmol/L or under treatment for type 2 diabetes mellitus.

### 2.4. Statistical Analysis

Data were recorded with the Epidata 3.1 database, and SPSS16.0 statistical software (SPSS Inc., Chicago, IL, USA) was used for statistical analysis. The demographic characteristics of the elderly were described in terms of rate and percentage. The chi-square test was used to compare the disability rate among the elderly with different demographic characteristics. The graph is used to show the disability rates of ADL, six items of PADL, and eight items of IADL at different ages. To obtain the relationship between ADL, PADL, IADL and its 14 items and age, the trend chi-square test was used to analyze whether there was a trend between them. Binary logistic regression was used to analyze the influencing factors of disability rate in the elderly. The independent variables were gender, age, marital status, education level, number of chronic diseases, smoking or not, alcohol use, and whether they had MetS. The dependent variables were ADL, PADL, IADL, and 14 items (with/without disability). The results of the binary logistic regression analysis were expressed in OR with a confidence interval of 95%. The test level was 0.05 on both sides.

## 3. Results

### 3.1. Demographic Characteristics

A total of 2300 elderly people agreed to participate in this study. The sample had the following characteristics: 58.7% females (1350 out of 2300); the youngest and oldest were 60 and 104 years old, respectively (average age: 70.32 ± 7.93 years), those aged 60~ years accounted for 53.2% (1350 out of 2300), 70~ years 37.2% (855 out of 2300), 80~ years 5.0% (116 out of 2300), and 90 years old and above accounted for 4.6% (106 out of 2300). The ethnic group is dominated by the Zhuang people, accounting for 81.3% (1869 out of 2300) of the sample. The majority of the sample was illiterate, accounting for 41.3% (1038 out of 2300). Those who were married accounted for 68.0% (1564 out of 2300). Those who were widowers accounted for 8.8% (203 out of 2300), widows accounted for 23.1% (532 out of 2300). Most of the elderly comprised farmers, accounting for 93.3% (2147 out of 2300), while the non-smoking and non-drinking respondents accounted for 83.3% (1917 out of 2300) and 76.7% (1763 out of 2300) of the sample, respectively (Table 1).

### 3.2. Disability Rates of ADL, PADL, and IADL in the Elderly People with Different Characteristics

The overall disability rate of ADL was 43.4% (998 out of 2300). A statistically significant difference was observed in ADL disability rates among the following variables: gender, age, occupation, marital status, educational level, whether or not the person was smoking and drinking, and whether the person had MetS (*p* < 0.05). The disability rate of females was higher than that of males. With age, the elderly had a higher disability rate. The disability rate in marriage was lower than that in non-marriage. As the level of education declined, the disability rate of elderly gradually increased. The incidence of elderly with smoking, drinking, and MetS was higher than that of those without smoking, drinking, and MetS.

The disability rate of PADL was 11.6% (266 out of 2300). Statistically significant differences were observed in the PADL disability rates among age, marital status, number of chronic disease, and whether with MetS (*p* < 0.05). The disability rate in marriage was lower than that in non-marriage, and the disability rate was higher with the decline of education. In addition, as the number of chronic diseases increased, the disability rate increased gradually. Finally, the disability rate of the elderly with MetS was higher than that without MetS.

The disability rate of IADL was 42.4% (976 out of 2300). The disability rate among different characteristics was the same as that of ADL. Except for ethnic groups and the number of chronic disease, the disability rates among other features were statistically significant (*p* < 0.05) (Table 1).

### 3.3. Trends of Disability Rates among Different Ages in ADL, PADL, IADL, and Their Items

**Figure 1:**
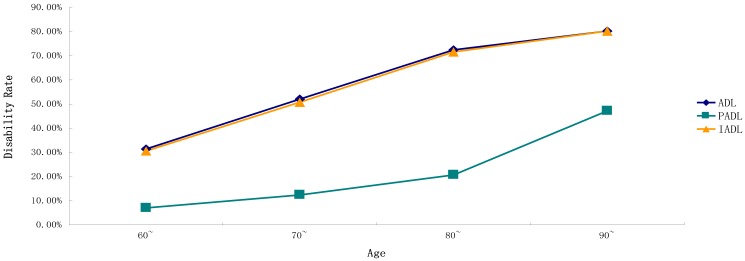
Disability rates of ADL, PADL, and IADL among the elderly at different ages.

**Figure 2:**
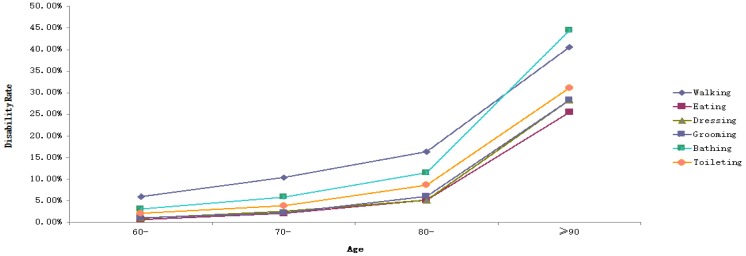
Disability rates of six items of PADL in the elderly at different ages.

**Figure 3:**
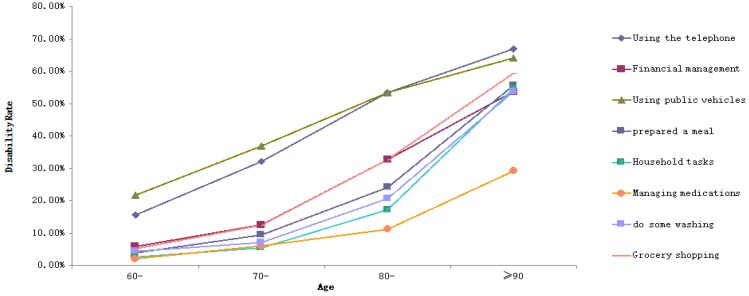
Disability rates of eight items of IADL in the elderly at different ages.

The results of the chi-square test for trends showed that, as age increased, the disability rates of ADL, IADL, PADL, and their 14 items gradually increased as well (*p* < 0.05) (Table 2). As revealed in Figure 1 and Table 2, the trends of ADL and IADL disability rates were consistent and at a relatively high level. The disability rates of ADL and IADL rose gradually from 30.0% in the age of 60 years to 80.2% in the age of 90 and above. While the disability rate of PADL was lower than those of ADL and IADL, its disability rate rose gradually from 7.0% in the age of 60 years to 47.2% in the age of 90 and above. With age, the disability rates of walking and bathing in the six items of PADL were higher than those of other items (Figure 2). Their disability rates rose gradually from 9.7% and 6.2% in the age of 60 to 40.6% and 44.4% in the age of 90 and above, respectively. The disability rate of eating was the lowest, gradually increasing from 2.6% in the age of 60 to 25.5% in the age 90 and above. The disability rates of using public vehicles and the telephone in the eight items of IADL were higher than those of other items. Their disability rates rose gradually from 21.8% and 15.6% in the age of 60 to 64.2% and 67.0% in the age of 90 and above, respectively (Figure 3).

### 3.4. Binary Logistic Analysis of the Influencing Factors of ADL, PADL, IADL, and 14 Items in the Elderly

As shown in Table 3, the influencing factors of ADL disability were gender (OR = 0.579, 95%CI = 0.441–0.759), age (OR = 2.270, 95%CI = 1.867–2.759; OR = 4.719, 95%CI = 2.998–7.429; OR = 6.249, 95%CI = 3.667–10.648), educational level (OR = 2.844, 95%CI = 2.076–3.897; OR = 1.677, 95%CI = 1.246–2.230), and MetS (OR = 1.298, 95%CI = 1.044–1.613). Compared with ADL, the influencing factor of PADL disability was lack of gender, educational level; and the influencing factor of IADL disability was lack of whether the subject had metabolic syndrome. The possible influencing factors of the 14 items of disability are detailed here. Age was the influencing factor for all items of disability (total of 14 items). The items that might be influenced by gender were as follows: eating (OR = 2.488, 95%CI = 1.147–5.397), dressing (OR = 2.235, 95%CI = 1.096–4.557), grooming (OR = 2.466, 95%CI = 1.206–5.045), using public vehicles (OR = 0.444, 95%CI = 0.329–0.599), preparing a meal (OR = 1.766, 95%CI = 1.130–2.761), performing household tasks (OR = 1.776, 95%CI = 1.059–2.980), and taking medication (OR = 1.739, 95%CI = 1.002–3.020) (total of six items). Educational level may have been an influencing factor for using the telephone (OR = 4.422, 95%CI = 2.880–6.791; OR = 2.054, 95%CI = 1.354–3.117), doing financial management (OR = 2.593, 95%CI = 1.515–4.441), using public vehicles (OR = 2.738, 95%CI = 1.661–3.404; OR = 1.631, 95%CI = 1.159–2.296), doing some washing (OR = 2.113, 95%CI = 1.115–4.005; OR = 2.273, 95%CI = 1.265–4.087), and grocery shopping (OR = 1.915, 95%CI = 1.114–3.293) (total of five items). Alcohol use might be the influence factor of eating (OR = 0.395, 95%CI = 0.164–0.953), bathing (OR = 0.520, 95%CI = 0.297–0.908), using the telephone (OR = 0.686, 95%CI = 0.489–0.961), preparing a meal (OR = 0.537, 95%CI = 0.333–0.865), performing household tasks (OR = 0.491, 95%CI = 0.278–0.866), doing some washing (OR = 0.598, 95%CI = 0.364–0.982), grocery shopping (OR = 0.517, 95%CI = 0.325–0.820).

The number of chronic diseases might be an influence factor for walking (OR = 2.723, 95%CI = 1.806–4.104; OR = 4.431, 95%CI = 2.954–6.645), bathing (OR = 1.654, 95%CI = 1.108–2.668; OR = 4.168, 95%CI = 2.509–6.922), toileting (OR = 2.071, 95%CI = 1.153–3.719), preparing a meal (OR = 1.765, 95%CI = 1.164–2.675; OR = 4.313, 95%CI = 2.772–6.710), performing household tasks (OR = 2.552, 95%CI = 1.547–4.212), taking medication (OR = 1.818, 95%CI = 1.102–3.000; OR = 3.394, 95%CI = 1.985–5.803), doing some washing (OR = 2.368, 95%CI = 1.531–3.665), and grocery shopping (OR = 1.883, 95%CI = 1.319–2.687; OR = 2.749, 95%CI = 1.845–4.096) (total of eight items). The presence of MetS in an individual might be an influencing factor for walking (OR = 1.584, 95%CI = 1.153–2.175), grooming (OR = 2.010, 95%CI = 1.399–3.413), toileting (OR = 2.185, 95%CI = 1.399–3.413), doing financial management (OR = 1.468, 95%CI = 1.081–1.994), preparing a meal (OR = 1.871, 95%CI = 1.333–2.626), performing household tasks (OR = 2.408, 95%CI = 1.626–3.565), doing some washing (OR = 1.520, 95%CI = 1.066–2.166), and grocery shopping (OR = 1.576, 95%CI = 1.156–2.147) (total of eight items) Table 3. Association among gender, age, marital status, education, smoking, alcohol use, chronic disease, metabolic syndrome, and disability; Odds Ratios (OR 95%CI).

## 4. Discussion

As the aging population continues to increase, maintaining the functional capacity of older adults has become an urgent concern [20]. The occurrence of ADL and IADL disabilities among the elderly is a complex process resulting from a combination of factors in medicine, sociology, psychology, and behavioral science [21]. The results of the current study showed that the disability rates of ADL, PADL, and IADL among the elderly were 43.4%, 11.6%, and 42.4%, respectively. The ADL disability rate in the current study is higher than that of Lima-Costa’s findings for Brazilian elderly (30.1%) [14] and Yin De-ting’s findings (41.0%) for Chinese elderly [15], but lower than Downer’s findings (44.0%) for Mexican and Mexican American adults aged 80 and over [13].

The IADL disability rate in the present study is higher than Shimada’s findings (26.7%) for 10,885 community-dwelling older adults aged 65 and enrolled in the NCGG-SGS [22]. The trends of ADL and IADL disability rates were consistent at a higher level, whereas PADL has a relatively low disability rate; both findings are consistent with the results of other researchers [17,23]. These findings might be related to the characteristics of PADL and IADL. PADL reflects the self-care ability of the elderly, whereas IADL reflects the elderly’s ability to live independently. The disability rate of IADL is higher than that of PADL, indicating that most elder people can take care of themselves, but they cannot live independently. This situation is similar to the research results of Jing Rui et al. [24]. This similarity can be attributed to the fact that the elderly who participated in the present survey came from remote rural areas and, as such, their educational level was generally low. Except for the above findings, this study found that gender, age, education level, and whether someone had MetS were the influencing factors of ADL disability. Age, number of chronic diseases, and whether someone had MetS were the influencing factors of PADL disability. Gender, age, and education level were the influencing factors of IADL disability. The results of the study about the influencing factors of disability are similar to those of other studies [25,26,27,28]. Previous research suggested that age, chronic disease, being female, having low economic income, and having low education level were considered as risk factors for disability. The above findings reveal that the disability of the elderly in the County is similar to the situation in other places, thereby suggesting that the local health administration can learn from the mature experiences of other places in designing future interventions.

Logistic regression analysis results showed that gender may be the influencing factor of ADL and IADL disability in the elderly, as females were more likely to encounter ADL and IADL disabilities than males. This finding is consistent with the results of past studies [6,29,30]. A study of 9694 older people by Lina Mad [6] found that females’ disability rate was 1.3 times than that of males. In the study of elderly people aged 65 and above, Chalise et al. found that the rate of disability in females IADL was 50.0%, which was significantly higher than that of males’ at 26.0% [29]. The possible reason was that elder females who participated in the study were mostly illiterate, and in rural areas, they were rarely associated with the outside world. However, gender differences in three items (preparing a meal, performing household tasks, and taking medication) in the IADL were reversed. This finding is consistent with that of a Nara study in Japan, which found that the rate of instrumental daily life impairment in males was higher than that of females [31]. The reasons might be related to local labor habits: local males are often responsible for physical activities outside the home and females are generally responsible for housework and child care at home. Thus, males are more likely to have disabilities than females. The probability that males or females are more likely to be disabled is different from the total ADL and IADL rates and in the above three items. Hence, this requires further investigation and subsequent follow-ups.

The trend chi-square test showed that the rate of disability increased with age. Logistic regression analysis results also indicated that age was the influencing factor for disability, as all the results (ADL, PADL, IADL, and 14 items) showed that the older the age was, the higher the OR value was. This finding is consistent with many studies [6,32,33,34]. In a study of the global burden of disease, Salomon et al. [35] found positive correlation between years of disability and life expectancy. Suzuki’s study of disability in Japan discovered that, although the functional capabilities of older people living in the Japanese community have improved over the past two decades, as age increased, the daily functional ability of the elderly worsened [20]. Three possible factors can explain this. First, in terms of research site, the elderly involved in the survey are mainly doing farming activities in the rural areas. Second, with the increase in age, various organic or functional changes occur in the elderly, and the prevalence of chronic diseases has thus has increased. Third, given the high proportion of empty-nest elderly people in rural areas, psychological problems, such as loneliness and depression, are more common among this population. In addition, walking is recognized as a predictor for the onset of ADL disability in the elderly [36]. Among the six items of PADL in this study, walking has the highest rate of disability, which also increases with age. Further, this finding supports the notion that the rate of disability increases with age. The reasons for the highest disability rate of using the telephone may be that, first, local economic conditions are poor, and some elderly people have never left the area. Mobile phones are new tools for them, and because they rarely interact with the outside world, they might have certain difficulties in using mobile phones. Second, many elderly people have declining hearing and vision as they age, which adds difficulty in making calls in daily life. Regarding the use of public vehicles, the local public vehicle is special, and tricycles are different from buses in cities. Many elderly people seldom go outside and if they do need to go out, they must have better ability in their physical coordination to get on and off vehicles. Many of them have difficulties in completing daily activities because the body function and coordination are declining. Hence, proposing measures allowing the elderly to maintain good daily living functions is an important and urgent task.

Meanwhile, educational level also served as an influencing factor of ADL and IADL. Five of the eight items of IADL showed that the lower the education level was, the higher the incidence of disability. Scholars believe that the high frequency of ADL disability is related to low education level [37,38,39,40]. The results in this study may have been influenced by the fact that most of the elderly in this study are illiterate, and such situation could have affected the activities of IADL, such as using the telephone, doing financial management, and using public vehicles. In addition, elder people with lower education levels might have less attention to physical health, not to mention the prevention of chronic disease.

In this study, although the results of multiple factors failed to conclude that alcohol consumption was not a possible influencing factor for the occurrence of ADL, PADL and IADL, it was concluded that alcohol consumption could be influencing factor for seven items. From the OR value, someone with alcohol use were less likely to occur disability than someone without alcohol use. Human activity and cognitive function were affected by alcohol use [41]. Studies have shown that alcohol can cause cognitive impairment because large drinkers have lower prefrontal cortex volume, activity, and oxygenation [42]. But other studies have found that moderate drinking not only slows cognitive decline in women [43], but also improves cognitive performance in older people [44]. Other studies have found moderate drinking did not affect or decrease the incidence of dementia and cognitive impairment [45]. The results of this study suggested that alcohol use might play a role in alleviating functional decline in certain daily activities. It was similar to the results of Stampfer et al. [43], it needs further research to prove it.

Studies have shown that the number of chronic diseases is associated with impaired ADL, and the more chronic diseases there are, the higher the rate of ADL disability would be [46,47,48]. Elderly people with high blood pressure [49], diabetes, or cerebrovascular disease [50] are susceptible to ADL or IADL. In this study, 65.7% of the elderly had one or more chronic diseases. This is consistent with the results of a recent census in the United States, which concluded that about 66.5% of older adults with one or more disabilities have difficulty in moving [51]. Logistic regression analysis showed that the probability of PADL disability in the elderly with one or two chronic diseases was 2.625 and 4.431 times higher than that of elderly without chronic diseases, respectively. The results of this study did not show a relationship between chronic disease and ADL or IADL disability. However, the results for five items showed that, with the increase in the number of chronic diseases, the possibility of disability in the elderly gradually increased. These items included walking and bathing in PADL and preparing a meal, taking medication, and grocery shopping in IADL. Except for taking medication, the other four items had similar characteristics, as the elderly needed to move their bodies within a large area. This finding is consistent with the results of Remillard et al. [52], who found that adults with long-term movement disorders must face multiple challenges related to ADLs/IADLs as they age.

Studies have also found that MetS is significantly related to the ADL and IADL of the elderly living in the community [53,54,55]. However, other studies have shown that MetS is associated with mobility restrictions but not related to the disability of ADL or IADL in older Mexican Americans [56]. The findings of the present study indicated that the elderly with MetS were more likely to have ADL and PADL disability than those without MetS. Although the overall IADL did not enter the regression equation, part of the IADL items entered the equation. Of all the PADL items, three items entered the regression equation: walking, grooming, and toileting. Meanwhile, of all the items in the IADL, five items entered the equation: doing financial management, preparing a meal, performing household tasks, doing some washing, and grocery shopping. The above results suggest that we should focus on the occurrence of MetS in this population. In the intervention measures, elderly people without MetS should do regular physical examinations, so that their metabolic values fall within the normal range, thus reducing the probability of physical disability.

This study has the following highlights. To the best of our knowledge, this is the first study to analyze the influencing factors of disability among the elderly in the county (Guangxi Province, China). Second, many current studies have analyzed the disability of the elderly but only by analyzing the overall ADL, PADL, IADL and few have studied each item in greater detail. In comparison, this study analyzed the risk factors for disability from ADL, PADL, IADL and each activity (14 items) to explore the possible factors of disability among the elderly living in the mountain areas. The study provides a scientific basis by offering basic data for further interventions, which can allow the local elderly to have a better quality of life. Third, for certain influencing factors, although multivariate analysis failed to show certain factors were risk factors for ADL, PADL, or IADL disability, a risk factor was indicated in certain specific activities (14 items reflected).

Several aspects, however, need further attention. First, as this survey is a cross-sectional study, it cannot determine the causal relationship between influencing factors and disability. Second, as the data were self-reported, individual bias may have influenced the information. Third, the number of samples in this study was not large enough. For this reason, enlarging the number of samples is needed to increase the credibility and extensibility of the results. This study is the baseline survey of our project, and we aim to do a follow-up study on the elderly who have participated in the survey every three years in the future.

## 5. Conclusions

Among the elderly in a County in Guangxi Province, China, an increase of age also increases the likelihood of being disability in terms of ADL, PADL, and IADL. Gender, age, educational level, the number of chronic diseases, and whether someone has MetS might be the influencing factors of disability. For some functions of daily life, alcohol use might play a role in relieving the decline in functional, further researches should be made to confirm it. 

## Figures and Tables

**Table 1:** Baseline characteristics of 2300 participants with and without incident ADL, PADL, and IADL disability.

Item	All Participants (n,%)	ADL (*n* = 998)			PADL (*n* = 266)			IADL (*n* = 976)		
		Impairment (*n*,%)	χ^2^	*p* Value	Impairment (*n*,%)	*χ^2^*	*p* Value	Impairment (*n*,%)	χ^2^	*p* Value
Gender			160.392	<0.001		0.416	0.519		167.684	<0.001
Male	950 (41.3%)	264 (27.8%)			105 (11.1%)			252 (26.5%)		
Female	1350 (58.7%)	734 (54.4%)			161 (11.9%)			724 (53.6%)		
Age			195.903	<0.001		165.98	<0.001		196.725	<0.001
60~	1223 (53.2%)	384 (31.4%)			86 (7.0%)			374 (30.6%)		
70~	855 (37.2%)	445 (52.0%)			106 (12.4%)			434 (50.8%)		
80~	116 (5.0%)	84 (72.4%)			24 (20.7%)			83 (71.6%)		
90~	106 (4.6%)	85 (80.2%)			50 (47.2%)			85 (80.2%)		
Ethnic			0.898	0.638		5.467	0.065		1.874	0.392
Han	317 (13.8%)	134 (42.3%)			49 (15.5%)			127 (40.1%)		
Zhuang	1869 (81.3%)	810 (43.3%)			205 (11.0%)			795 (42.5%)		
Others	114 (5.0%)	54 (47.4%)			12 (10.5%)			54 (47.4%)		
Occupation			31.777	<0.001		2.22	0.136		31.073	<0.001
Farmer	2147 (93.3%)	965 (44.9%)			254 (11.8%)			944 (44.0%)		
Others	153 (6.7%)	33 (21.6%)			12 (7.8%)			32 (20.9%)		
Marital status			77.550	<0.001		25.15	<0.001		81.273	<0.001
Divorced/Widowed	736 (32.0%)	417 (56.7%)			121 (16.4%)			412 (56.0%)		
Married	1564 (68.0%)	581 (37.1%)			145 (9.3%)			564 (36.1%)		
Level of education			223.042	<0.001		5.826	0.054		236.274	<0.001
Literacy	1038 (41.3%)	612 (59.0%)			134 (12.9%)			605 (58.3%)		
Primary school	797 (34.7%)	295 (37.0%)			92 (11.5%)			289 (36.3%)		
Junior high school and above	465 (20.2%)	91 (19.6%)			40 (8.6%)			82 (17.6%)		
Current smoking			65.144	<0.001		0.288	0.295		66.161	<0.001
Without	1917 (83.3%)	904 (47.2%)			228 (11.9%)			886 (46.2%)		
With	381 (16.7%)	94 (24.7%)			38 (10.0%)			90 (23.6%)		
Alcohol use			66.522	<0.001		3.481	0.062		69.965	<0.001
Without	1763 (76.7%)	847 (48.0%)			216 (12.3%)			832 (47.2%)		
With	537 (23.3%)	151 (28.1%)			50 (9.3%)			144 (26.8%)		
Chronic conditions			3.322	0.345		35.134	<0.001		2.975	0.396
0	788 (34.3%)	340 (43.1%)			55 (7.0%)			337 (42.8%)		
1	995 (43.3%)	449 (45.1%)			120 (12.1%)			436 (43.8%)		
2	390 (17.0%)	160 (41.0%)			67 (17.2%)			154 (39.5%)		
3	127 (5.5%)	49 (38.6%)			24 (18.9%)			49 (38.6%)		
Metabolic Syndrome			8.000	0.005		17.352	<0.001		6.002	0.014
Without	1764 (76.7%)	737 (41.8%)			177 (10.0%)			724 (41.0%)		
With	536 (23.3%)	261 (48.7%)			89 (16.6%)			252 (47.0%)		

**Table 2:** Trends of disability rates among different age groups in ADL, PADL, IADL, and their items.

Item	Total	60~	70~	80~	≥90	χ^2^	*p* Value
	*n* (%)	*n* (%)	*n* (%)	*n* (%)	*n* (%)		
ADL	998 (43.4%)	384 (31.4%)	445 (52.0%)	84 (72.4%)	85 (80.2%)	191.989	<0.001
PADL	266 (11.6%)	86 (7.0%)	106 (12.4%)	24 (20.7%)	50 (47.2%)	138.334	<0.001
IADL	976 (42.4%)	374 (30.6%)	434 (50.8%)	83 (71.6%)	85 (80.2%)	193.711	<0.001
Walking	224 (9.7%)	73 (6.0%)	89 (10.4%)	19 (16.4%)	43 (40.6%)	114.135	<0.001
Eating	59 (2.6%)	8 (0.7%)	18 (30.6%)	6 (5.2%)	27 (25.5%)	158.834	<0.001
Dressing	70 (3.0%)	12 (1.0%)	22 (2.6%)	6 (5.2%)	30 (28.3%)	158.023	<0.001
Grooming	69 (3.0%)	13 (1.1%)	19 (2.2%)	7 (6.0%)	30 (28.3%)	76.049	<0.001
Bathing	142 (6.2%)	38 (3.1%)	50 (5.8%)	13 (11.5%)	41 (44.4%)	153.701	<0.001
Toileting	102 (4.4%)	26 (2.1%)	33 (3.9%)	10 (8.6%)	33 (31.1%)	134.112	<0.001
Using the telephone	599 (26.0%)	191 (15.6%)	275 (32.2%)	62 (53.4%)	71 (67.0%)	222.151	<0.001
Financial management	275 (12.0%)	72 (5.9%)	108 (12.6%)	38 (32.8%)	57 (53.8%)	234.731	<0.001
Using public vehicles	712 (31.0%)	266 (21.7%)	316 (37.0%)	62 (53.4%)	68 (64.2%)	144.48	<0.001
Preparing a meal	216 (9.4%)	48 (3.9%)	81 (9.5%)	28 (24.1%)	59 (55.7%)	275.191	<0.001
Performing household tasks	157 (6.8%)	32 (2.6%)	47 (5.5%)	20 (17.2%)	58 (54.7%)	302.783	<0.001
Taking medication	122 (5.3%)	26 (2.1%)	52 (6.1%)	13 (11.2%)	31 (29.2%)	130.917	<0.001
Doing some washing	195 (8.5%)	54 (4.4%)	61 (7.1%)	24 (20.7%)	57 (53.8%)	232.805	<0.001
Grocery shopping	272 (11.8%)	64 (5.2%)	107 (12.5%)	38 (32.8%)	63 (59.4%)	287.099	<0.001

**Table 3:** Association among gender, age, marital status, education, smoking, alcohol use, chronic disease, metabolic syndrome, and disability; Odds Ratios (OR 95%CI).

**Item**	**Gender**	**Age**			**Marital Status**
	**Female = Reference**	**60~ = Reference**			**Married = Reference**
	**Male OR (95%CI)**	**70~OR (95%CI)**	**80~OR (95%CI)**	**90~OR (95%CI)**	**NO Married OR (95%CI)**
ADL	0.579 (0.441–0.759)	2.270 (1.867–2.759)	4.719 (2.998–7.429)	6.249 (3.667–10.648)	1.193 (0.971–1.464)
PADL	1.267 (0.853–1.882)	1.815 (1.328–2.480)	3.864 (2.251–6.634)	17.948 (10.464–30.786)	1.065 (0.780–1.455)
IADL	0.573 (0.436–0.753)	2.221 (1.825–2.703)	4.626 (2.945–7.266)	6.356 (3.726–10.843)	1.212 (0.987–1.489)
Walking	1.094 (0.714–1.676)	1.813 (1.296–2.537)	3.602 (2.001–6.485)	17.407 (9.909–30.578)	0.955 (0.681–1.339)
Eating	2.488 (1.147–5.397)	2.811 (1.193–6.623)	7.207 (2.318–22.406)	47.160 (17.762–125.216)	1.445 (0.732–2.852)
Dressing	2.235 (1.096–4.557)	2.313 (1.119–4.780)	4.733 (1.658–13.513)	39.150 (16.154–90.096)	1.321 (0.712–2.448)
Grooming	2.466 (1.206–5.045)	1.857 (0.896–3.849)	5.334 (1.970–14.443)	37.118 (15.777–87.327)	1.246 (0.664–2.340)
Bathing	1.652 (0.982–2.779)	1.773 (1.134–2.772)	4.107 (2.019–8.355)	25.002 (13.240–47.212)	1.159 (0.758–1.772)
Toileting	1.686 (0.926–3.070)	1.782 (1.040–3.053)	4.520 (2.015–10.140)	24.885 (12.229–50.638)	1.282 (0.779–2.112)
Using the telephone	0.726 (0.527–1.001)	2.362 (1.884–2.962)	4.550 (2.958–6.999)	6.597 (4.069–10.695)	1.263 (1.007–1.584)
Financial management	1.297 (0.854–1.968)	1.936 (1.401–2.676)	5.095 (3.135–8.280)	9.631 (5.821–15.936)	1.711 (1.267–2.312)
Using public vehicles	0.444 (0.329–0.599)	2.037 (1.655–2.508)	3.473 (2.280–5.290)	4.802 (3.008–7.665)	1.110 (0.896–1.374)
Preparing a meal	1.766 (1.130–2.761)	2.392 (1.630–3.509)	8.393 (4.772–14.762)	41.319 (22.916–74.499)	1.067 (0.746–1.525)
Performing household tasks	1.776 (1.059–2.980)	2.054 (1.278–3.300)	8.208 (4.275–15.761)	54.054 (28.304–103.231)	1.231 (0.801–1.891)
Taking medication	1.739 (1.002–3.020)	2.681 (1.637–4.391)	5.267 (2.508–11.062)	19.318 (9.774–38.181)	1.292 (0.832–2.006)
Doing some washing	1.452 (0.914–2.308)	1.470 (0.996–2.170)	5.071 (2.882–8.920)	23.393 (13.342–41.016)	1.235 (0.856–1.784)
Grocery shopping	1.173 (0.774–1.780)	2.319 (1.660–3.241)	7.674 (4.651–12.664)	25.275 (14.722–43.392)	1.281 (0.939–1.748)
**Item**	**Education Level**			**Smoking**	**Alcohol Use**
	**High = Reference**			**Without Smoking = Reference**	**Without Alcohol Use = Reference**
	**Low OR (95%CI)**		**Average OR (95%CI)**	**With Smoking OR (95%CI)**	**With Alcohol use OR (95%CI)**
ADL	2.844 (2.076–3.897)		1.667 (1.246–2.230)	0.835 (0.610–1.143)	0.847 (0.648–1.107)
PADL	1.154 (0.714–1.868)		1.310 (0.857–2.001)	0.983 (0.628–1.539)	0.724 (0.486–1.078)
IADL	3.116 (2.260–4.297)		1.834 (1.360–2.472)	0.849 (0.617–1.168)	0.834 (0.636–1.094)
Walking	1.088 (0.650–1.821)		1.204 (0.762–1.903)	0.870 (0.530–1.429)	0.810 (0.527–1.244)
Eating	1.822 (0.582–5.700)		2.300 (0.807–6.557)	1.336 (0.554–3.221)	0.395 (0.164–0.953)
Dressing	1.899 (0.669–5.391)		2.375 (0.914–6.170)	1.163 (0.510–2.656)	0.450 (0.203–1.000)
Grooming	1.896 (0.663–5.421)		2.486 (0.954–6.476)	0.978 (0.419–2.284)	0.463 (0.208–1.030)
Bathing	1.441 (0.735–2.826)		1.549 (0.849–2.825)	1.073 (0.588–1.959)	0.520 (0.297–0.908)
Toileting	0.900 (0.416–1.945)		1.270 (0.655–2.463)	1.027 (0.525–2.008)	0.605 (0.326–1.122)
Using the telephone	4.422 (2.880–6.791)		2.054 (1.354–3.117)	0.827 (0.549–1.245)	0.686 (0.489–0.961)
Financial management	2.593 (1.515–4.441)		1.341 (0.799–2.252)	0.878 (0.529–1.458)	0.714 (0.461–1.105)
Using public vehicles	2.738 (1.661–3.404)		1.631 (1.159–2.296)	1.112 (0.775–1.595)	0.780 (0.576–1.056)
Preparing a meal	1.748 (0.974–3.137)		1.649 (0.972–2.796)	0.896 (0.527–1.524)	0.537 (0.333–0.865)
Performing household tasks	1.052 (0.530–2.090)		1.407 (0.767–2.580)	0.735 (0.387–1.398)	0.491 (0.278–0.866)
Taking medication	1.666 (0.821–3.381)		1.333 (0.696–2.552)	0.998 (0.528–1.887)	0.656 (0.369–1.168)
Doing some washing	2.113 (1.115–4.005)		2.273 (1.265–4.087)	1.017 (0.586–1.764)	0.598 (0.364–0.982)
Grocery shopping	1.915 (1.114–3.293)		1.463 (0.880–2.433)	0.891 (0.528–1.504)	0.517 (0.325–0.820)
**Item**	**Number of Chronic Conditions**				**Metabolic Syndrome**
	**0 = Reference**				**Without Mets = Reference**
	**1 OR (95%CI)**			**≥2 OR (95%CI)**	**With Mets OR (95%CI)**
ADL	1.208 (0.980–1.490)			1.066 (0.830–1.369)	1.298 (1.044–1.613)
PADL	2.625 (1.800–3.828)			4.431 (2.954–6.645)	1.690 (1.257–2.272)
IADL	1.161 (0.941–1.434)			1.036 (0.805–1.332)	1.240 (0.996–1.543)
Walking	2.723 (1.806–4.104)			4.813 (3.110–7.451)	1.584 (1.153–2.175)
Eating	1.052 (0.539–2.053)			1.811 (0.859–3.820)	1.792 (0.974–3.299)
Dressing	1.406 (0.764–2.591)			1.741 (0.852–3.556)	1.654 (0.944–2.900)
Grooming	1.363 (0.734–2.530)			1.783 (0.870–3.654)	2.010 (1.153–3.504)
Bathing	1.654 (1.018–2.668)			4.168 (2.509–6.922)	1.467 (0.981–2.193)
Toileting	1.495 (0.889–2.515)			2.071 (1.153–3.719)	2.185 (1.399–3.413)
Using the telephone	0.957 (0.757–1.209)			0.788 (0.590–1.053)	1.202 (0.943–1.533)
Financial management	1.040 (0.761–1.422)			0.983 (0.668–1.446)	1.468 (1.081–1.994)
Using public vehicles	1.084 (0.871–1.349)			0.895 (0.685–1.168)	1.199 (0.957–1.502)
Preparing a meal	1.765 (1.164–2.675)			4.313 (2.772–6.710)	1.871 (1.333–2.626)
Performing household tasks	1.233 (0.777–1.956)			2.552 (1.547–4.212)	2.408 (1.626–3.565)
Taking medication	1.818 (1.102–3.000)			3.394 (1.985–5.803)	1.492 (0.978–2.277)
Doing some washing	1.326 (0.891–1.974)			2.368 (1.531–3.665)	1.520 (1.066–2.166)
Grocery shopping	1.883 (1.319–2.687)			2.749 (1.845–4.096)	1.576 (1.156–2.147)
